# Reducing attention bias toward negative emotional stimuli with transcranial random noise stimulation: a randomized, double-blind, sham-controlled, crossover study

**DOI:** 10.1038/s41598-025-28306-w

**Published:** 2025-12-12

**Authors:** Daisuke Sawamura, Miaowen Duan, Yuji Inagaki, Ryuji Saito, Kazufumi Okada, Akihiro Watanabe, Harukazu Tohyama, Takaya Maeyama, Susumu Yoshida, Mitsunori Miyazaki, Naoya Hasegawa, Koichi Yokosawa, Yumie Ono

**Affiliations:** 1 Department of Rehabilitation Science, Faculty of Health Sciences, Hokkaido University, Sapporo, 060-0812 Hokkaido Japan; 2 Department of Physiotherapy, Faculty of Medicine, Dentistry and Health Sciences, University of Melbourne, Melbourne, 3010 Australia; 3 Graduate School of Health Sciences, Hokkaido University, Sapporo, 060- 0812 Japan; 4 Department of Rehabilitation Sciences, Health Sciences University of Hokkaido, Tobetsu, 061-0293 Japan; 5 Data Science Center, Promotion Unit, Institute of Health Science Innovation for Medical Care, Hokkaido University Hospital, Sapporo, 060- 8648 Japan; 6 Department of Integrative Physiology, Graduate School of Biomedical and Health Sciences, Hiroshima University, Hiroshima, 734-8553 Japan; 7 Department of Electronics and Bioinformatics, School of Science and Technology, Meiji University, Kawasaki, 214-8571 Japan; 8 Department of Health Sciences, Faculty of Health Sciences, Hokkaido University, Hokkaido 060-0812 Sapporo, Japan

**Keywords:** Attention bias, Dorsolateral prefrontal cortex, Transcranial random noise stimulation, Transcranial direct current stimulation, Cognitive neuroscience, Emotion, Neuronal physiology

## Abstract

Excessive attention bias interferes with daily life and contributes to various psychiatric conditions. Previous studies have demonstrated that anodal transcranial direct current stimulation (tDCS) applied to the left dorsolateral prefrontal cortex (DLPFC) can enhance attentional control and reduce negative emotional bias. However, little is known about the effects of transcranial random noise stimulation (tRNS) on attention bias. This study aimed to investigate whether tRNS reduces attention bias toward negative emotional stimuli compared to tDCS, for which functional evidence has been established. This randomized, double-blind, crossover, sham-controlled study enrolled 32 healthy right-handed men. The emotional Stroop task was used to assess attention bias. The participants experienced all three stimulation conditions: tRNS, tDCS, or sham, with each condition separated by a 7-day washout interval and administered in a random order. tRNS to the left DLPFC significantly reduced attention bias toward negative stimuli compared with sham (corrected *p* = 0.047, Cohen’s d = 0.72), whereas no significant difference was observed between tRNS and tDCS. Positive correlations were observed between the effects of each stimulation condition and attention bias in the sham condition in the tRNS and tDCS groups; however, these associations did not exceed the empirical null distribution determined by permutation testing. Thus, the modulatory effects of tRNS on attentional control may not strictly depend on baseline bias magnitude, suggesting its potential applicability in both healthy and clinical populations characterized by excessive attention bias.

**Clinical Trial Registration**: This study was registered in the University Hospital Medical Information Network Clinical Trial Registry in Japan (UMIN000052202) on 09/13/2023; https://center6.umin.ac.jp/cgi-open-bin/ctr_e/ctr_his_list.cgi?recptno=R000059574.

## Introduction

Attention bias refers to the phenomenon of being more sensitive to and focusing longer on specific stimuli, particularly threatening stimuli, than on neutral stimuli^1,2^. Excessive attention bias toward threat-relevant stimuli is associated with the onset, progression, and maintenance of several affective disorders^3,4^. Attention bias is broadly shared among individuals with depression, social anxiety disorder, generalized anxiety disorder, social phobia, and post-traumatic stress disorder. Healthy individuals with high trait anxiety also exhibit attention bias^1,2,5,6^.

The neural basis of attention bias has been broadly reported to involve the prefrontal-amygdala pathway, which is a part of the emotion generation-regulation network. An imbalance in this network leads to the occurrence of attention bias^7,8^. The amygdala, a subcortical limbic structure, plays a role in the early detection of threat-relevant stimuli, triggering alertness and attentional focus, and is involved in the generation of negative emotions, such as fear and disgust^2,9,10^. Increased amygdala activation amplifies the responses to threat-relevant stimuli and promotes attention bias^2,7,8^. Conversely, the prefrontal cortex regulates attention to threat stimuli and controls negative associations^11–13^. Of these, the left dorsolateral prefrontal cortex (DLPFC) plays an important role in the avoidance of excessive engagement through attentional control or disengagement, indirectly inhibiting the activity of subcortical limbic structures, such as the amygdala, through top-down control by regulating the ventromedial prefrontal cortex (VMPFC)^14–17^. Neuroimaging studies have confirmed increased amygdala and decreased prefrontal cortex activity in patients with anxiety disorders^2,10,18^. In patients with depression, decreased left DLPFC activity and decreased functional connectivity weaken control over the amygdala and VMPFC, increasing sensitivity to threat-relevant stimuli and attention bias^10,18^. Thus, attention bias is thought to result from the interaction between excessive amygdala and reduced DLPFC activity. Accordingly, increasing the excitability of the left DLPFC and the efficiency of its neural network with the amygdala is predicted to improve top-down control over the amygdala, thereby reducing attention bias relevant to mental health and behavioral regulation.

Transcranial electrical stimulation (tES) is a noninvasive brain stimulation technique that modulates cortical excitability by applying a weak current through electrodes, thereby inducing neural plasticity changes. Transcranial direct current stimulation (tDCS) is a representative tES technique that modulates cortical and subcortical excitability by altering polarity-specific membranes. Noninvasive brain stimulation methods targeting attention bias primarily involve transcranial magnetic stimulation and anodal tDCS applied to the left prefrontal cortex. Both techniques have been reported to reduce attention bias toward negative emotional stimuli during stimulation. However, the effects of tDCS on attention bias demonstrate considerable heterogeneity among studies and small effects^16^. Factors contributing to the variability in effects of tDCS include psychopathological state, emotional valence and arousal level of the emotional task, the specific cortical area targeted, and other related stimulus parameters, including intensity, duration, and timing^16^. Additionally, inter-individual variability in responsiveness to tDCS may contribute significantly to these findings^19,20^.

However, no studies have investigated the effects of transcranial random noise stimulation (tRNS) on emotional attention bias. tRNS is a novel form of tES characterized by alternating currents delivered at random intensities, which presumably amplify neural responses via stochastic resonance^21^. Previous studies reported that tRNS applied to the left DLPFC improves creativity^22^, attentional control^23^, working memory^24^, and inhibitory control^25^ and reduces pain^26^. Some studies indicated that tRNS has more prominent effects than tDCS^23,24^. High-frequency tRNS (HF-tRNS) applied to the VMPFC improves emotion recognition^27^, but this effect is absent when HF-tRNS targets the left DLPFC^28,29^. Thus, the left DLPFC appears to be critical for attentional control of emotions rather than for recognizing emotional stimuli, a precursor to emotional regulation^2,30^. Moreover, individuals with anxiety disorders or high trait anxiety exhibit particularly pronounced responses to tDCS of the left DLPFC, suggesting that tRNS may elicit similar neuromodulatory sensitivity^31–34^. Taken together, these findings indicate that tRNS applied to the left DLPFC may be especially effective at modulating emotional attention bias.

This study aimed to investigate whether tRNS over the left DLPFC, a key region for modulating attention bias, enhances regulation of attention bias toward negative emotional stimuli. It also aimed to compare the efficacy of tRNS with that of tDCS applied to the same region. We hypothesized that tRNS would more effectively reduce attention bias to negative stimuli than tDCS. Moreover, we anticipated that participants with higher trait anxiety and greater attention bias would show the largest improvements.

## Methods

### Trial design and participants

This randomized, double-blind, crossover, sham-controlled study recruited 32 right-handed young men (18–30 years) with moderate or high anxiety levels over 15 months (October 2023 through December 2024). The decision to focus on male participants was informed by a previous meta-analysis^35^ that highlighted sex differences in subjective and physiological responses to negative emotional stimuli. Handedness and anxiety were assessed using the Edinburgh Handedness Inventory (EHI)^36^ and the State-Trait Anxiety Inventory-JYZ (STAI-JYZ), respectively^37^. The required sample size was estimated using G power 3.1^38^ based on a previous randomized crossover study assessing the effects of tRNS on attentional network efficiency^23^. We adopted partial *η*^*2*^ (*η*^*2*^_*p*_) of the main effect based on a repeated measures analysis of variance (rmANOVA). The sample size estimate for achieving a 0.95 statistical power at a significance level of α = 0.05 and a given effect size (f) of 0.402 was calculated by the effect size of the main effect for stimulation *η*^*2*^_*p*_ of 0.139^23^; this resulted in a suggested sample size of *n* = 27. A conservative adjustment of 20% was applied to the estimated sample size to accommodate potential dropouts and outliers, yielding a final sample size of *n* = 32.

The exclusion criteria were (1) a low trait anxiety score (≤ 30% of the normative score: male individuals score ≤ 42) on the STAI-JYZ, (2) an EHI score of < 70 points, (3) a history of neurological or psychiatric disorders, and (4) insufficient safety assessed using a safety questionnaire for transcranial electrical stimulation (metal implants, pacemakers, history of epilepsy, and pregnancy)^39^.

Independent experimenters randomly assigned all participants to receive all three stimulations (tRNS, tDCS, and sham) in a random order according to a computer-generated stratified randomization list with stratification factors (age and STAI-JYZ). The stimulation order was fully randomized and counterbalanced across the participants. The interval between each session was set as at least 7 days to consider any carry-over effects of stimulations^40^ and habituation to the task. All participants and experimenters, except those responsible for random assignments, were blinded to stimulation conditions.

All participants provided written informed consent before inclusion in the study and received financial compensation. The study was conducted in accordance with the Declaration of Helsinki and Consolidated Standards of Reporting Trials guidelines. This study was approved by the Institutional Review Board of the Faculty of Health Sciences at Hokkaido University (approval number: 23–71) and prospectively registered on 09/13/2023 in the University Hospital Medical Information Network Clinical Trial Registry in Japan (UMIN000052202).

### Experimental procedure

**Fig. 1:**
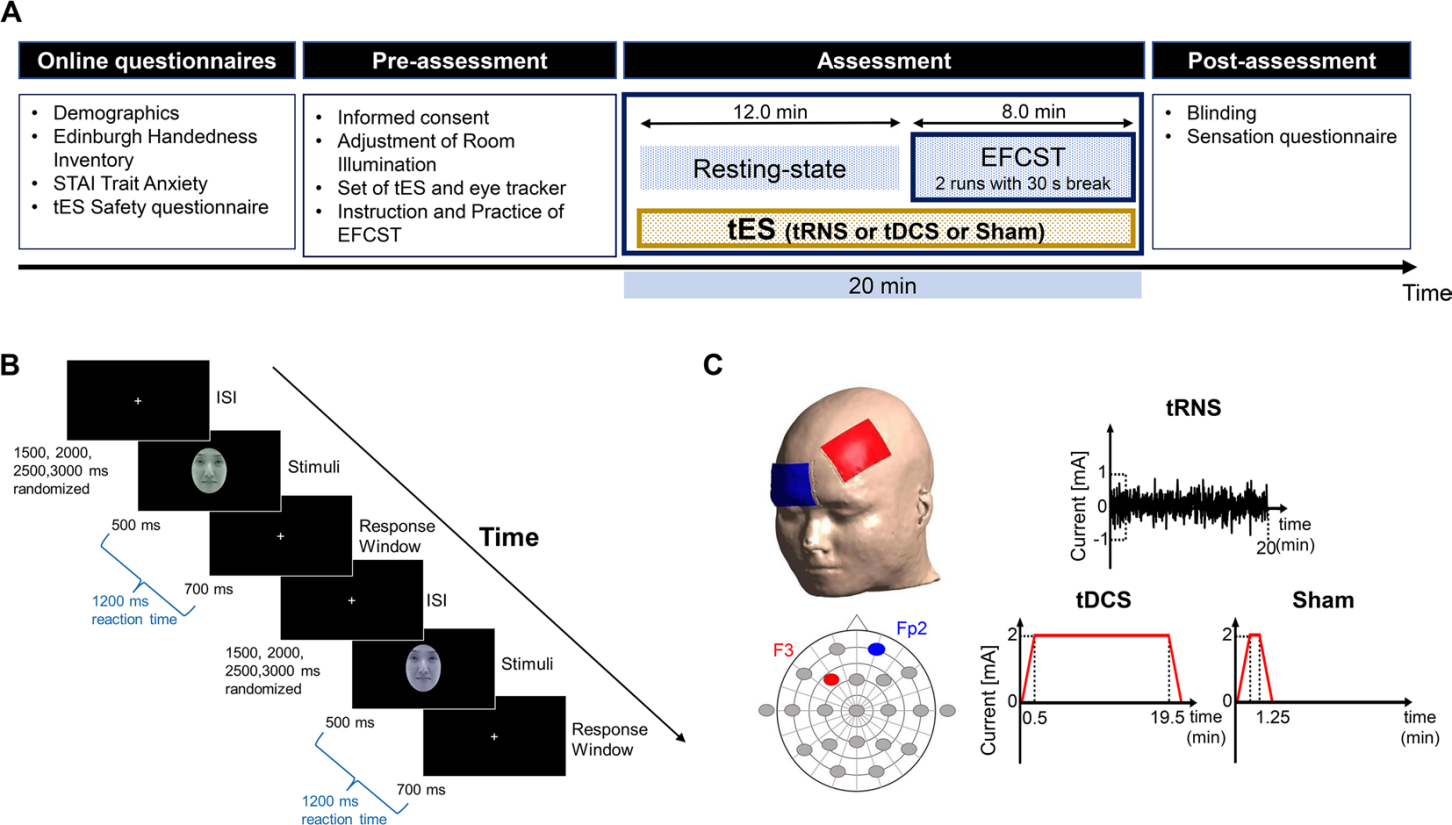
Schematic representation of the experimental procedure, emotional face-color Stroop task, and tES setting. **(a)** Time course of experimental procedure. The experimental procedure in each session consisted of three phases: pre-assessment, assessment, and post-assessment. Any type of three stimulations was applied in each session during the assessment phase and participants performed the EFCST for the last eight minutes. (**b)** Time series of EFCST. A fixation cross is presented randomly for 1500 ms, 2000 ms, 2500 ms, or 3000 ms, followed by a face image in one of four colors displayed for 500 ms. The response time consists of 500 ms during the presentation of the face image and the following 700 ms with the fixation cross, totaling 1200 ms. Facial emotional stimuli were created using a sample image of the facial expression database from the supplemental materials (Fujimura & Umemura, 2018). We obtained permission to use only sample images of neutral facial expressions from the National Institute of Advanced Industrial Science and Technology.**(c)** Electrode placement and three types of stimulation protocol. The anode and cathode are respectively placed over the left DLPFC (F3) and the right orbitofrontal cortex (Fp2) according to the international EEG 10/20 system. tRNS delivers an alternating current with a random frequency and intensity. tDCS delivers a direct electrical current with a constant intensity. EFCST: emotional face-color Stroop task; tDCS: transcranial direct current stimulation; tES: transcranial electrical stimulation; tRNS: transcranial random noise stimulation.

The overall procedure is illustrated in Fig. 1a. Prior to participation, all participants completed online questionnaires (demographic, EHI, STAI-JYZ, and safety questionnaire for transcranial electrical stimulation), and eligibility was confirmed. Each session consisted of three phases: pre-assessment, assessment, and post-assessment; all sessions were completed on a single day.

In the pre-assessment phase, participants received an explanation of the overall procedure and tasks, completed a written informed consent form, and underwent screening of their daily health conditions. They also completed a practice session of 24 trials to learn how to perform the task and minimize the learning effect.

In the assessment phase, the participants sat on a chair and received one of three stimulations (tRNS, tDCS, or sham) to the left DLPFC in a random order. In the initial 12 min online, they received stimulation while in a resting state. Subsequently, they performed an 8-minute main task consisting of 2 blocks of 64 trials, with a total of 128 trials. To ensure thorough blinding, each experimenter independently delivered the stimuli and assessed participants’ performances.

During the post-assessment phase, we evaluated stimulation blindness and tES-related sensations to verify trial validity and ensure participant safety and tolerability. A structured questionnaire assessed tES sensations and blinding^41^. For blinding, participants were asked, “Do you believe you received real or placebo stimulation?” with three options: (1) “real,” (2) “placebo,” or (3) “I do not know.” Those selecting “I do not know” were then prompted to make their best guess from either “real” or “placebo,” following the procedure of Bang et al.^42^.

### Attention bias task

Attention bias was assessed using the emotional face-color Stroop task (EFCST) programmed in PsychoPy^43^, employing facial images from the AIST Facial Expression Database^44^ (Fig. 1b). Previous studies have shown that disgusted faces capture attention more strongly than other emotional stimuli in individuals with moderate-to-high anxiety^45–47^. Moreover, within the AIST database, disgusted faces have the lowest valence among all facial images, making them especially suitable for eliciting attention bias^44^. Accordingly, we selected disgusted faces as negative emotional stimuli, neutral faces as baseline, and angry faces as control stimuli to prevent habituation. As angry faces served only as controls, trials featuring them were excluded from the analysis.

The experimental material consisted of 32 neutral, 16 angry, and 16 disgusted facial images, with equal proportions of male and female faces. Each facial image was paired with one of four colors (red, blue, yellow, or green). Participants were asked to respond to the color of the colored facial images (disgust, anger, and neutral) presented after fixation by pressing a key (“D,” “F,” “J,” and “K”) on a keyboard. Finger placement for key pressing was also instructed: “D,” “F,” “J,” and “K” with the left middle, left index, right index, and right middle fingers, respectively. The correspondence between the colors and keys was counterbalanced. To prevent habituation to the stimuli, a fixation cross was presented for randomly selected durations of 1500, 2000, 2500, or 3000 ms in each trial, with equal probability (Fig. 1b).

Each run of the EFCST consisted of 64 trials in 225 s; 2 runs, totaling 128 trials, were performed with a 30-second break between them, resulting in a total duration of 8 min. The participants sat on a chair 60 cm away from a 23-inch monitor, with a resolution of 1920 × 1080 pixels, and kept both their hands on the keyboard. After the presentation began with a fixation cross at the center of the screen, a colored emotional face with a visual angle of 5.3° × 4.0° was presented for 500 ms. Subsequently, a response time window of 700 ms was provided for the participants to respond to the corresponding color.

In the EFCST, an incorrect key press, no response, and a reaction time (RT) shorter than 200 ms and longer than 1200 ms were defined as errors and were removed from the analyses. RTs longer or shorter than the mean ± 3 standard deviations were also excluded per participant from the analysis. The main outcome measure, attention bias, was calculated by subtracting the RTs of neutral facial images from those of disgusted facial images.

To ensure the perception of the target stimuli, the ratio of stimulus gazing during the task was measured using an eye-tracker device (X60, Tobii Technology, Sweden) for each participant throughout the assessment phase. The ratio of stimulus gazing was calculated as the percentage of time that the participant’s gaze remained within a facial image ellipse with a visual angle of 5.3° × 4.0° for the total duration of the target presentation.

### Transcranial electrical stimulation

Stimulation was delivered using a DC-STIMULATOR PLUS (NeuroConn GmbH, Ilmenau, Germany) with a pair of 5 × 7 cm rubber electrodes soaked in 0.9% saline solution, yielding a current density of 0.057 mA/cm². For all stimulation conditions, a pair of electrodes was placed over the left DLPFC (F3) and right supraorbital cortex (Fp2), as defined by the international 10–20 electroencephalography (EEG) system. A schematic of the electrode configuration and all stimulations is shown in Fig. 1c.

We based this electrode placement on previous studies indicating that higher stimulation intensity and longer duration over the left DLPFC enhance modulation of cognitive functions, including attention bias, in healthy and clinical populations^48^. Specifically, 2-mA tRNS for 20 min over the left DLPFC improved attentional control and working memory more prominently than tDCS^23,24^. Accordingly, the intensity and duration for both tRNS and tDCS were set to 2 mA and 20 min, respectively. With tRNS, a 2-mA intensity denotes the peak-to-peak amplitude (0–2 mA), indicating that the stimulation fluctuates randomly around 0 mA with a maximum excursion of ± 1 mA. A high-frequency tRNS (100 to 640 Hz), corresponding to the upper range of physiologically observed human brain oscillations^49^, was employed based on prior evidence indicating its superior neuromodulatory efficacy compared to low-frequency tRNS (0–100 Hz)^21,40^.

In the tRNS and tDCS conditions, a 30-second gradual ramping up to 2 mA and a 30-second gradual ramping down were applied at the beginning and end of the 20-minute online session. In the sham condition, a 30-second gradual ramping up to 2 mA, 15 s of 2-mA stimulation, and 30 s of gradual ramping down were applied during the initial 75 s of the 20-minute session, with no current for the remainder of the period. This blinding procedure helped minimize the differential sensations between the active and sham conditions. Impedances during stimulation were maintained below 5.0 kΩ in all stimulation conditions.

### Statistical analyses

Blinding assessments, sensation questionnaire responses, and target-gazing rates were analyzed using one-way rmANOVA, the Kruskal–Wallis test, or the chi-square test.

Attention bias was analyzed using one-way rmANOVA. We first confirmed normality using the Shapiro–Wilk test and assessed sphericity via Mauchly’s test; if sphericity was violated (*p* < 0.05), degrees of freedom were adjusted using the Greenhouse–Geisser correction. Partial eta squared (η²ₚ) served as the effect-size measure. To examine the influence of session order, we compared models with and without session number as a covariate using the Akaike Information Criterion (AIC), retaining the simpler model if inclusion of session did not lower AIC by more than 5 points^50^. When rmANOVA reached significance, post-hoc comparisons applied Bonferroni correction (multiplying each p-value by three), and we calculated Cohen’s d and the standard deviation ratio (SDR) to quantify effect magnitude and variability.

To assess the relationships between stimulus-related benefits and both (1) attention bias in the sham condition and (2) anxiety scores, we performed Pearson’s correlation analyses with a within-subject label-flip permutation test (10,000 iterations) to preserve within-subject dependencies. We then computed the partial correlation coefficients to control for the shared influence of anxiety and attention bias in the sham condition on these associations.

In addition, we performed simple linear regression to quantify the magnitude of this relationship. We then applied a general linear model to each stimulation condition to compare regression slopes, thereby characterizing the benefits of each condition.

All statistical analyses were performed using SPSS version 26.0 for Windows (IBM Corp., Armonk, NY, USA) and R (version 4.x; R Foundation for Statistical Computing, Vienna, Austria). The α level was set at 0.05.

## Results

### Baseline characteristics, sensation questionnaires, blinding assessment, and gazing rate

**Fig. 2:**
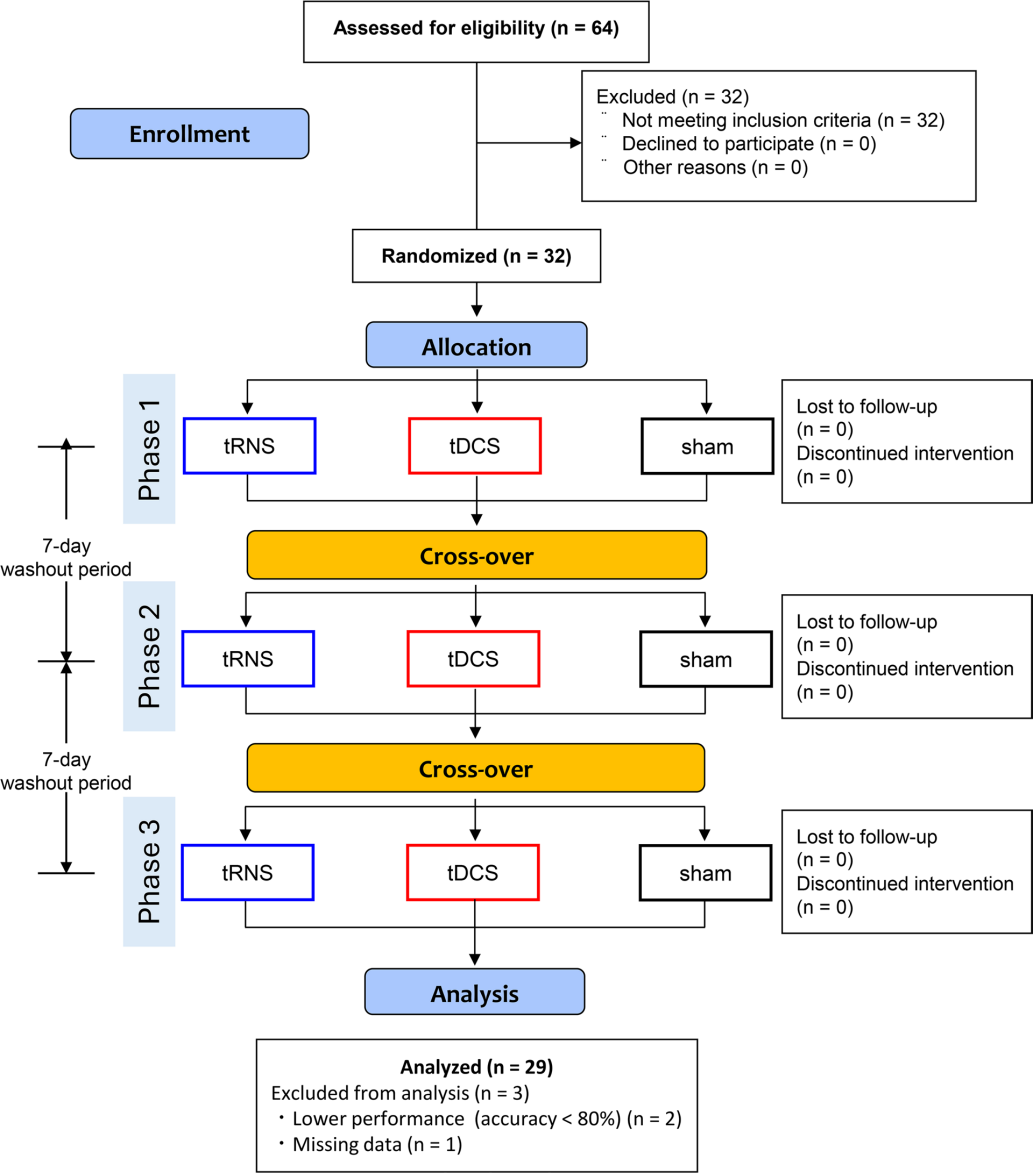
Consolidated Standards of Reporting Trials flow diagram. The flow diagram provides the number of participants and demonstrates the procedural steps to the final data analysis. tRNS: transcranial random noise stimulation; tDCS: transcranial direct current stimulation.

Figure 2 shows the Consolidated Standards of Reporting Trials flow diagram. Of the 64 applicants, 32 were excluded for not meeting the inclusion criteria (trait anxiety score ≤ 42). The final sample comprised 29 right-handed healthy young adults (age 18–28 years; mean age 20.7 ± 1.7 years; STAI trait anxiety score: 51.28 ± 6.63; mean EHI score 95.99 ± 8.82). Regarding the STAI trait anxiety scores, 10 participants (34.5%) had moderate anxiety levels (43–48 points), and 19 participants (65.5%) had high anxiety levels (49 points and above).

Table 1 shows the results of the sensation questionnaires, blinding, and gaze rates. There were no significant differences among the three stimulus conditions. tRNS exhibited blinding and discomfort values that were nearly equivalent to those of the sham condition. A common high gazing rate (> 95%) across groups was observed, which ensured stimulus input.

**Table 1:** Comparison of blinding, discomfort, and fixation rate across the three conditions.

	tRNS (*n* = 29)	tDCS (*n* = 29)	Sham (*n* = 29)	*p*-value	Statistics
Blinding				0.149	χ^2^=3.81
Felt stimulated n (%)	15 (51.7%)	22 (75.9%)	16 (58.6%)		
Felt non-stimulated n (%)	14 (48.3%)	7 (24.1%)	13 (41.4%)		
Discomfort Median (IQR)					
Tingling pain	1.0 (0.0–1.0)	1.0 (1.0–2.0)	1.0 (0.0–1.0)	0.155	χ^2^=9.27
Pain	0.0 (0.0–0.0)	0.0 (0.0–0.5)	0.0 (0.0–0.0)	0.317	χ^2^=4.72
Fatigue	1.0 (0.0–1.0)	0.0 (0.0–1.0)	0.0 (0.0–1.0)	0.629	χ^2^=4.36
Warmth/Heat	0.0 (0.0–0.0)	0.0 (0.0–0.0)	0.0 (0.0–0.0)	0.901	χ^2^=0.21
Burning	0.0 (0.0–0.0)	0.0 (0.0–0.0)	0.0 (0.0–0.0)	0.37	χ^2^=4.28
Itching	0.0 (0.0–0.0)	0.0 (0.0–0.0)	0.0 (0.0–0.0)	0.27	χ^2^=5.17
Smell of metal	0.0 (0.0–0.0)	0.0 (0.0–0.0)	0.0 (0.0–0.0)	0.36	χ^2^=2.02
Target gazing rate (%)*	96.50%	96.30%	95.60%	0.115	F = 2.26

IQR: interquartile range; * denotes the results of 26 samples.

### Modulation effects on attention bias

**Fig. 3:**
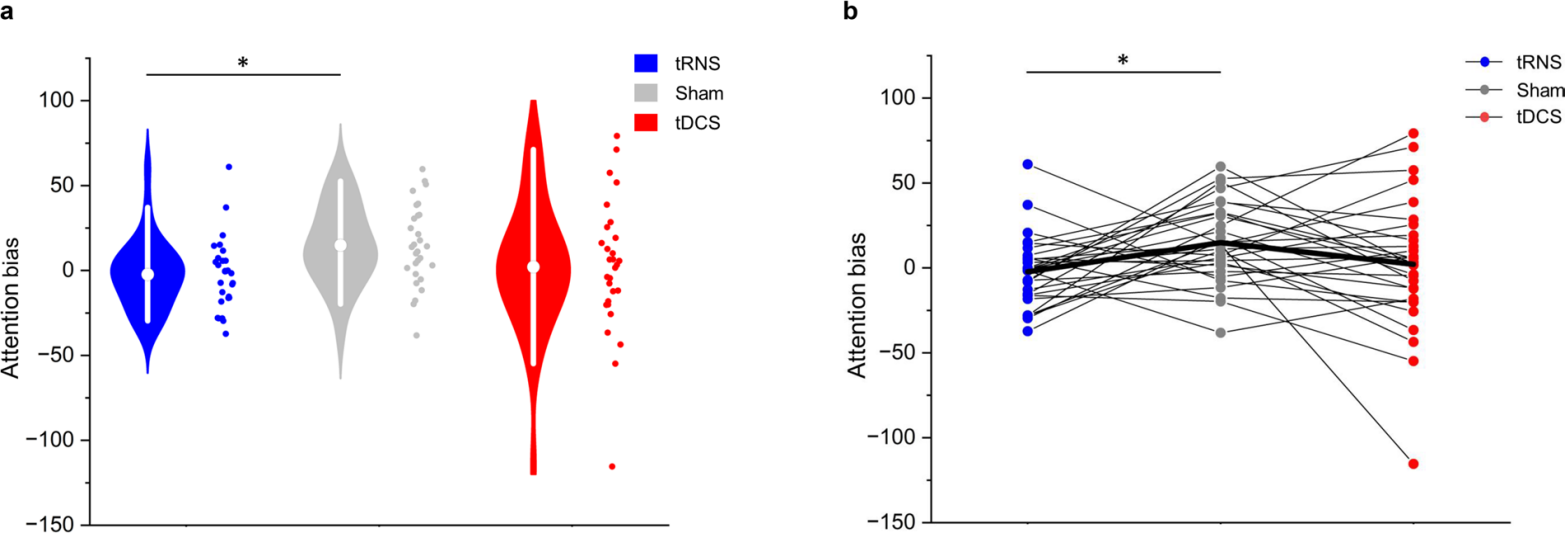
Effects of each stimulation condition on the attention bias scores. **(a**) Violin plot with individual attention bias scores. White circles and error bars within Violin plots indicate the mean and 95% confidence interval. (**b**) Individual performance across three stimulation conditions. The bold lines indicate the mean attention score across three stimulation conditions. * Bonferroni-corrected *p* < 0.05.

Figure 3 shows the EFCST attention bias scores for all groups. Adding session number as a covariate did not reduce the AIC by more than 5 points (simple model AIC = 770.43, covariate model AIC = 767.78); therefore, we retained the simple model without the covariate for analysis.

The rmANOVA revealed a significant group effect (F(2,56) = 3.63, *p* = 0.033, *η*²_*p*_ = 0.115). A post-hoc test showed a significantly lower attention bias score in the tRNS condition than in the sham condition (Bonferroni corrected *p* = 0.047, Cohen’s d = 0.72). However, no significant differences were observed in other comparisons (tDCS vs. sham: Bonferroni-corrected *p* = 0.276, Cohen’s d = 0.39; tDCS vs. tRNS: Bonferroni-corrected *p* = 1.00, Cohen’s d = 0.22). SDRs were 0.70 in tRNS and 1.10 in tDCS, respectively.

### Relationships among changes in attention bias, attention bias in the sham condition, and the STAI trait anxiety score

**Fig. 4:**
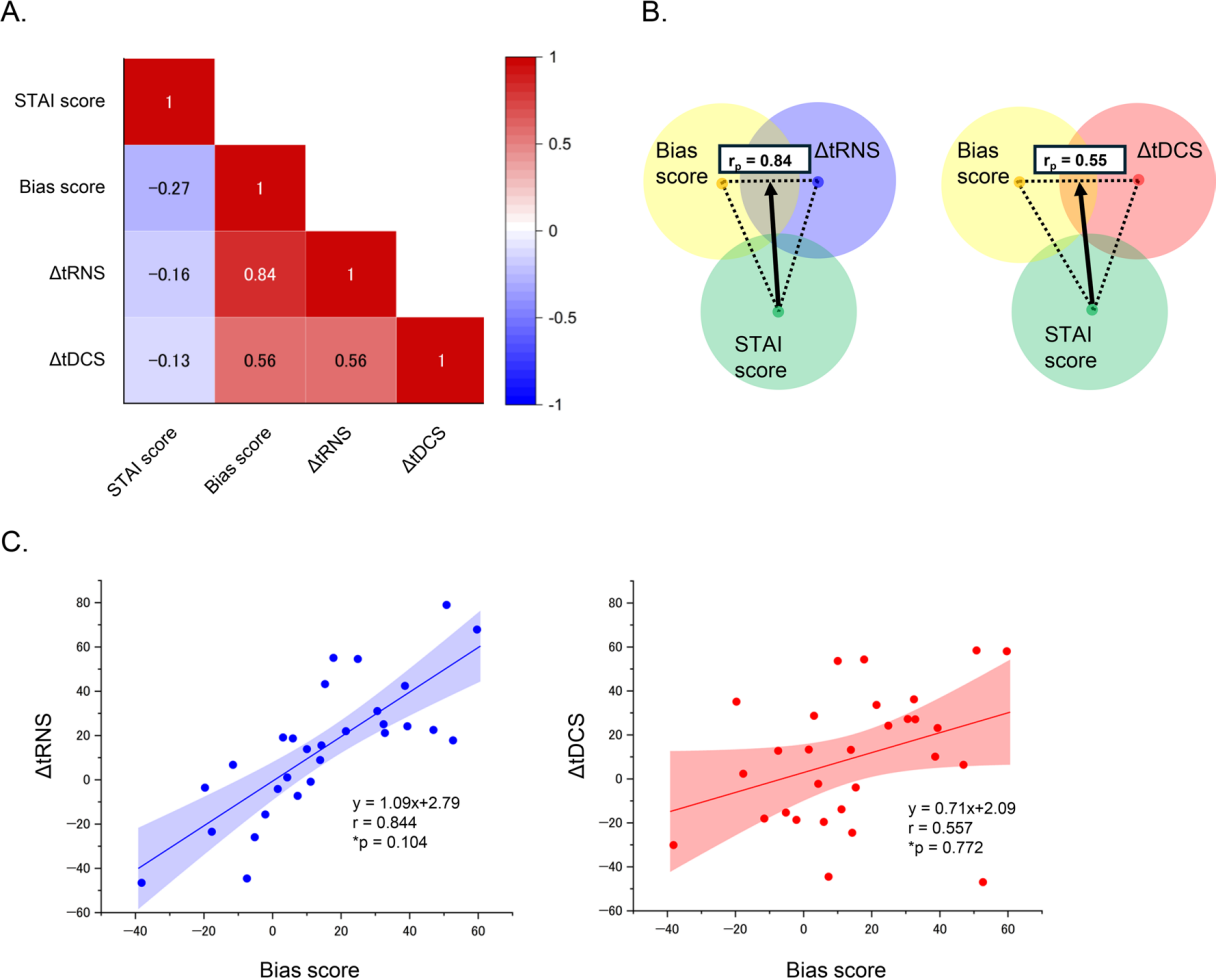
Overview of the correlations illustrating the relationships between the changes in attention bias, STAI trait anxiety score, and attention bias in the sham condition. **(A)** Correlation matrix of three indices, with the look-up table indicating the correlation coefficients. (**B)** Venn diagrams showing the relationships between three indices. The thick arrows indicate partial correlations between the baseline bias score and changes in attention bias in both tRNS and tDCS with the STAI trait anxiety score as a control variable. (**C)** Scatter plots depicting changes in attention bias for tRNS and tDCS against attention bias in the sham condition. The straight and curved lines represent the estimated regression lines and 95% confidence intervals, respectively. * indicates a permuted p-value.

Figure 4a illustrates the associations among the STAI trait anxiety score, baseline (sham) attention bias, and stimulation-induced changes in attention bias (Δ = active − sham). Positive associations were observed for both tRNS and tDCS; however, these did not reach statistical significance in the within-subject label-flip permutation test (tRNS: *r* = 0.844, permuted *p* = 0.104; tDCS: *r* = 0.557, permuted *p* = 0.772). No significant correlations were observed between the changes in attention bias and STAI trait anxiety scores for either tRNS (*r* = −0.158, *p* = 0.414) or tDCS (*r* = − 0.127, *p* = 0.513) (Fig. 4a). Similarly, attention bias in the sham condition did not correlate significantly with the STAI trait anxiety score (*r* = −0.269, *p* = 0.158). Figure 4b and c summarize the partial correlation analyses and corresponding scatter plots. The relationships between the changes in attention bias in both tRNS and tDCS and the baseline bias score, with the STAI trait anxiety score as the control variable, were almost identical in the direct and partial correlation analyses (tRNS: *r*_*p*_ STAI = 0.843; tDCS: *r*_*p*_ STAI = 0.547). Moreover, a simple linear regression analysis revealed significant positive associations between the changes in attention bias in both tRNS and tDCS and attention bias in the sham condition (changes in tRNS: *β* = 1.03, t(27) = 8.18, *p* < 0.001; changes in tDCS: *β* = 0.71, t(27) = 3.49, *p* = 0.002). Furthermore, general linear models, which aid in elucidating whether each tES had a different regression slope with respect to baseline performance, revealed no significant effect of stimulation condition (tRNS vs. tDCS × baseline performance interaction, F(1,54) = 2.417, *p* = 0.126, *η*^*2*^_*p*_ = 0.043).

## Discussion

This study examined the efficacy of tRNS on attentional bias toward negative stimuli in healthy individuals with moderate-to-high anxiety, aligning with recent consensus and position statements emphasizing transparency, reproducibility, and standardization^51^. Although no significant difference was observed between the tRNS and tDCS conditions, only tRNS significantly reduced attention bias relative to sham stimulation. To the best of our knowledge, this is the first study to directly compare tRNS and tDCS over the left DLPFC for modulating negative attentional bias. Blinding was successful overall, although participants in the tDCS condition reported slightly greater awareness of stimulation, and subjective discomfort was minimal or non-significant.

tRNS over the left DLPFC during stimulation significantly reduced attention bias to negative emotional stimuli and disgusted faces compared to the sham stimulation. Meanwhile, no significant effect of tDCS over the left DLPFC was observed. These findings align with previous evidence indicating that tRNS elicits more pronounced neuromodulatory effects on both neurophysiological and behavioral outcomes compared to tDCS^23,24,52–54^. Furthermore, a relatively small effect variability was confirmed, suggesting that tRNS has relative efficacy over tDCS in reducing attention bias toward negative emotional stimuli compared to tDCS.

The left DLPFC plays a critical role in regulating emotions and controlling attention bias to negative stimuli^14–17^. Studies focusing on the excitatory effects of tDCS and transcranial magnetic stimulation applied to the left DLPFC have shown reduced threat-related attention bias and improvement in anxiety states and antidepressant effects^33,55^. Evidence suggests that the activation of the left DLPFC suppresses amygdala activity, enhancing the ability to modulate attention bias toward threatening stimuli^34^.

The neurophysiological mechanism underlying the effects of tRNS on the left DLPFC is thought to involve activation of emotion regulation neural circuits centered around the amygdala. tRNS induces neuronal excitation across the cortex, increases neural noise through a stochastic resonance mechanism, and elicits broader cortical neuronal firing than tDCS^21,56^. This broader neuronal firing promotes the synchronization of neural activity across different brain regions, enhancing the transmission and distributed processing of the brain’s nonlinear systems, and thereby improving information-processing efficiency^56^.

Contò et al.^57^. have reported that tRNS in the bilateral parietal lobes (P3/P4) increases resting-state functional connectivity of the working memory-related network and dorsal and ventral attentional networks, compared with the tDCS and sham conditions, and shows a significant positive correlation between changes in task performance and changes in its connectivity. Wu et al.^58^. have also reported that tRNS in the bilateral temporal cortices enhances visuospatial task performance, has more generalized and dispersive effects across a broader range of brain areas, and increases resting-state functional connectivity of the frontoparietal and default mode networks compared to tDCS. Accordingly, these findings provide supporting evidence that tRNS enhances generalized and dispersive effects across a broader range of brain areas via the stochastic resonance mechanism. This may improve the functioning of the emotional regulation network responsible for top-down signal transmission from the DLPFC to the amygdala, thereby supporting our interpretation that tRNS enhances activation of the emotional regulation network, resulting in improved attention bias. However, long-term effects of tRNS remain inconsistent across studies^29,59^, highlighting the need for further systematic investigation to clarify its clinical utility and sustainability.

In this study, tDCS to the left DLPFC did not show a significant effect, compared with sham stimulation. tDCS enhances the cortical excitability in specific brain regions by applying a weak unidirectional electric current. One possible reason for this is that the participants in this study were healthy individuals with trait anxiety rather than clinical populations with anxiety disorders. In healthy individuals, significant reductions in prefrontal cortex activity, as observed in clinical populations, are not typically observed, and studies have reported variability in prefrontal cortex activity levels^2,60^. This may limit the benefits of tDCS in enhancing cortical excitability in the DLPFC. In fact, our findings demonstrated a higher SDR in tDCS than in tRNS, indicating a higher effective variability. Although the effects of tDCS on the left DLPFC have been confirmed in healthy individuals with trait anxiety^31,34^, these studies recruited participants with higher trait anxiety levels, which potentially led to more pronounced benefits.

Regarding the relationships among trait anxiety, attention bias, and changes in attention bias scores from baseline to either the tRNS or tDCS condition, no significant correlations were found in any of the analyses. One possible reason for these results is that the participants in this study were healthy individuals with trait anxiety ranging from moderate-to-high levels and were not in a pathological state. Previous studies have reported a close relationship between attention bias toward negative information and higher trait anxiety, demonstrating that participants with higher trait anxiety tend to show stronger attention biases^3,61^. However, multiple factors, including anxiety, depression, personality traits, and emotional disorders, contribute to attention bias^62–64^. Given these findings, the limited trait anxiety level of our participants was not the main factor that determined attention bias, and a complex interplay of multiple factors influenced this relationship, resulting in no significant correlation between attention bias and trait anxiety. Thus, tRNS has a consistent effect on improving attention bias regardless of trait anxiety level.

In contrast, although these relationships did not reach statistical significance in the within-subject permutation analysis, positive associations were observed between the changes in attention bias in both tRNS and tDCS and baseline attention bias, suggesting that participants with greater initial bias tended to show larger reductions following stimulation. The partial correlations controlling for trait anxiety yielded similar patterns, suggesting that these associations were not confounded by individual differences in anxiety.

Furthermore, no significant difference was observed in the regression slopes between tRNS and tDCS. However, it is important to note that the regression coefficient obtained from tRNS was close to 1.0, indicating that tRNS nearly eliminated the attention bias caused by emotional stimuli. Although this effect may be partly stimulus-specific (e.g., limited to disgusted facial images), it is consistent with previous evidence that tRNS produces broader and more potent cortical modulation than tDCS^57,58^. Taken together, these findings suggest that, although the baseline-dependent relationship requires further validation, tRNS may exert relatively strong modulatory effects on attentional control processes.

This study has some limitations. First, this study lacked neurophysiological evidence, such as EEG, fMRI, or autonomic measures, to corroborate the observed behavioral changes; future research should incorporate physiological metrics to more comprehensively assess attention bias. Second, although blinding was generally successful, participants reported slightly greater awareness of stimulation in the tDCS condition, potentially introducing expectancy effects that should be considered when interpreting group differences. Third, we measured only the immediate (online) effects of a single tES session to minimize habituation from repeated tasks; our pilot data confirmed that repeated assessments (baseline, online, and offline on the same day) induce a significant habituation effect that reduces attention bias. To mitigate this, we implemented a 7-day interval in the crossover design to accommodate both sustained stimulation effects and task habituation. Future studies should investigate offline effects using paradigms with reduced learning effects or other habituation controls. Finally, we recruited only young men with moderate-to-high trait anxiety. As tES efficacy may depend on anxiety level, future work should examine effects in individuals with very high or low anxiety and in clinical populations and include female participants to validate generalizability.

In conclusion, this study demonstrated significant modification effects of tRNS over the left DLPFC on attention bias toward negative stimuli. Although positive associations between the effects of each stimulation condition and attention bias in the sham condition were observed in both the tRNS and tDCS groups, these relationships did not reach statistical significance. These findings suggest that the modulatory effects of tRNS on attentional control may not strictly depend on baseline bias magnitude, supporting its potential applicability across both healthy and clinical populations characterized by heightened vulnerability. The results reported herein provide preliminary evidence for the clinical utility of tRNS as a neuromodulatory approach to mitigating excessive attention bias and should be verified through further replication.

## Data Availability

The datasets generated and/or analyzed in the current study are available from the corresponding author upon reasonable request.
